# Genetic Polymorphisms and Platinum-based Chemotherapy Treatment Outcomes in Patients with Non-Small Cell Lung Cancer: A Genetic Epidemiology Study Based Meta-analysis

**DOI:** 10.1038/s41598-017-05642-0

**Published:** 2017-07-17

**Authors:** Li-Ming Tan, Cheng-Feng Qiu, Tao Zhu, Yuan-Xiang Jin, Xi Li, Ji-Ye Yin, Wei Zhang, Hong-Hao Zhou, Zhao-Qian Liu

**Affiliations:** 1 Department of Clinical Pharmacology, Xiangya Hospital, Central South University, Changsha, 410008 P.R. China; 2 Department of Pharmacy, The First People’s Hospital of Huaihua City, Huaihua, 418000 P.R. China; 3 Institute of Clinical Pharmacology, Central South University, Hunan Key Laboratory of Pharmacogenetics, Changsha, 410078 P.R. China

## Abstract

Data regarding genetic polymorphisms and platinum-based chemotherapy (PBC) treatment outcomes in patients with NSCLC are published at a growing pace, but the results are inconsistent. This meta-analysis integrated eligible candidate genes to better evaluate the pharmacogenetics of PBC in NSCLC patients. Relevant studies were retrieved from PubMed, Chinese National Knowledge Infrastructure and WANFANG databases. A total of 111 articles comprising 18,196 subjects were included for this study. The associations of genetic polymorphisms with treatment outcomes of PBC including overall response rate (ORR), overall survival (OS) and progression-free survival (PFS) were determined by analyzing the relative risk (RR), hazard ration (HR), corresponding 95% confidence interval (CI). Eleven polymorphisms in 9 genes, including *ERCC1* rs11615 (OS), rs3212986 (ORR), X*PA* rs1800975 (ORR), *XPD* rs1052555 (OS, PFS), rs13181 (OS, PFS), *XPG* rs2296147 (OS), *XRCC1* rs1799782 (ORR), *XRCC3* rs861539 (ORR), *GSTP1* rs1695 (ORR), *MTHFR* rs1801133 (ORR) and *MDR1* rs1045642 (ORR), were found significantly associated with PBC treatment outcomes. These variants were mainly involved in DNA repair (EXCC1, XPA, XPD, XPG, XRCC1 and XRCC3), drug influx and efflux (MDR1), metabolism and detoxification (GSTP1) and DNA synthesis (MTHFR), and might be considered as potential prognostic biomarkers for assessing objective response and progression risk in NSCLC patients receiving platinum-based regimens.

## Introduction

Lung cancer is a leading cause of cancer-associated death and substantially contributes to the heavy burden worldwide, with a dismal 5-year survival rate of 16.6%^1^. Among all primary lung cancers, non-small cell lung cancer (NSCLC) represents approximately 85% of cases. Chemotherapy remains the standard first-line treatment for almost 80% of NSCLC patients, of which platinum-based chemotherapy (PBC) is considered as the most efficacious option, especially for patients with an advanced stage of the disease^2, 3^. Unfortunately, PBC efficacy varies markedly across individuals. Besides clinical and pathologic features, genetic variation is considered as an important factor to influence the treatment efficacy and prognosis.

For decades, we have witnessed a growing interest in the pharmacogenomics field, and a tremendous amount of epidemiological evidence that gene polymorphisms could give rise to varying drug response has emerged. Many studies have reported the association of genetic factors, including genes related to DNA repair pathway, drug influx and efflux, drug metabolism and detoxification, DNA synthesis, cell cycle control and apoptosis, with PBC response and prognosis of patients^4–8^. The accumulation of pharmacogenomics findings calls for a more comprehensive systematic review and meta-analysis to summarize the evidence and to identify the general genetic associations among reported results. Some meta-analyses have studied the influences of certain genes on treatment outcomes of NSCLC patients receiving PBC. However, these findings including original studies are not always consistent, and no systematic review and meta-analysis covering all tested polymorphisms has been performed thus far.

The aim of this work is to identify the effects of all eligible genes in clinical prognosis of NSCLC patients receiving platinum-based treatment. A total of 24 single nucleotide polymorphisms (SNPs) of 12 genes (*ERCC1*, *XPA*, *XPC*, *XPD*, *XPG*, *XRCC1*, *XRCC3*, *GSTP1*, *MTHFR*, *RRM1*, *MDR1* and *CDA*) have been studied in our work. The impacts of these genetic variants on PBC efficacy in NSCLC patients were assessed by evaluating the objective response ratio (ORR), progression-free survival (PFS), and overall survival (OS). We think this comprehensive meta-analysis with robust evidence would fill the gap in the pharmacogenomics of platinum in NSCLC patients.

## Materials and Methods

### Search strategy, eligibility criteria and data extraction

We followed the principles proposed by the Human Genome Epidemiology Network (HuGeNet) HuGE Review Handbook of Genetic Association Studies^9^.

Relevant studies were searched in PubMed, Chinese National Knowledge Infrastructure (CNKI) and WANFANG databases. A two-step search strategy was implemented and last updated on January 31, 2016. First, the following three groups of keywords were used for searching in MEDLINE (via the PubMed gateway): platinum OR cisplatin OR carboplatin OR oxaliplatin OR nedaplatin, polymorphism OR SNP OR variant, NSCLC OR non-small cell lung cancer. Second, we used different combinations of the above terms for complementary searching. Besides, references cited in the retrieved papers were manually searched in case of missing relevant studies. Afterwards, we singled out the candidate genes that were eligible in our research, and the terms including a candidate gene’s official symbol and the three above-mentioned groups of keywords were used to perform a comprehensive search.

The studies included in the meta-analysis had to meet all the following inclusion criteria: (i) cancer should be confirmed as NSCLC; (ii) treatment regimens were platinum-based chemotherapies; (iii) studies provided primary outcomes of interest including ORR, PFS or OS. Studies met any one of the exclusion criteria listed below were excluded in our analysis: (i) studies without indispensable data such as genotypes, overall response rate (ORR), overall survival (OS), or progression-free survival (PFS); (ii) studies with other types of lung cancer such as small cell lung cancer (SCLC) included; (iii) reviews, case reports, and meta-analyses. (iv) studies based on cell lines and animal experiment.

All records were screened by three investigators independently (Tan, Qiu and Jin) with disagreement resolved by discussion. The following information was extracted from each of the eligible studies: first author, publication year, sample size, ethnicity, age, gender, stages of tumor, chemotherapeutic agents, SNPs and genotyping methods, treatment outcomes.

### Statistical analysis

We used the ORR as an indicator for PBC efficacy. Patients were classified into two groups: the responding group, which included complete and partial responders (CR and PR), and the non-responding group, which included subjects with stable or progressive diseases (SD and PD)^10^. RR and the corresponding 95% CI were used to assess the association between each genetic variant and the response of NSCLC patients treated with PBC. The hazard ratios (HR) and corresponding 95% CI were determined to evaluate OS and PFS. Three genotypic models commonly used in genetic association synopses were applied in this meta-analysis: heterozygous or homozygous variant versus wild type, heterozygous variant versus wild type and homozygous variant versus wild type.

Between-study variance, also known as heterogeneity, was evaluated by the chi-square-based Q test based on chi- square as well as I^2^. Q tests with *P* > 0.10 were considered with statistical significance. I^2^ described the proportion of variation originating from heterogeneity rather than within-study error, whose value varied from 0 to 100 percent and indicated different heterogeneity degrees. Heterogeneity could be accepted when I^2^ < 50% (0 < I^2^ < 25%: no heterogeneity; 25 < I^2^ < 50%: moderate heterogeneity). Sensitivity analysis and subgroup analysis were also applied to find the source of heterogeneity. Pooled RRs and HRs were calculated using the fixed-effects model when the heterogeneity was under the moderate degree or did not exist. Otherwise, the random-effects model was used. Moreover, the potential publication bias was assessed by statistical evaluation with Begg’s funnel plot and Egger’s linear regression test. The α level of significance was set at 0.05 unless noted otherwise.

In the end, we calculated the false positive report probability (FPRP) of statistically significant results to assess whether the findings were noteworthy^11^. The FPRP value was determined based on the P value, the prior probability for the association and statistical power. We set a stringent FPRP threshold of 0.20 and assigned a prior probability range of 0.1–0.001, and the statistical power was based on the ability to detect an OR of 1.5, with α equal to the observed p-value.

All statistical analyses were performed with STATA/SE.12.0 (StataCorp, College station, TX) and R (version 3.2.0, R Foundation for Statistical Computing, Vienna, Austria).

## Results

### Characteristics of Eligible Studies

**Figure 1:**
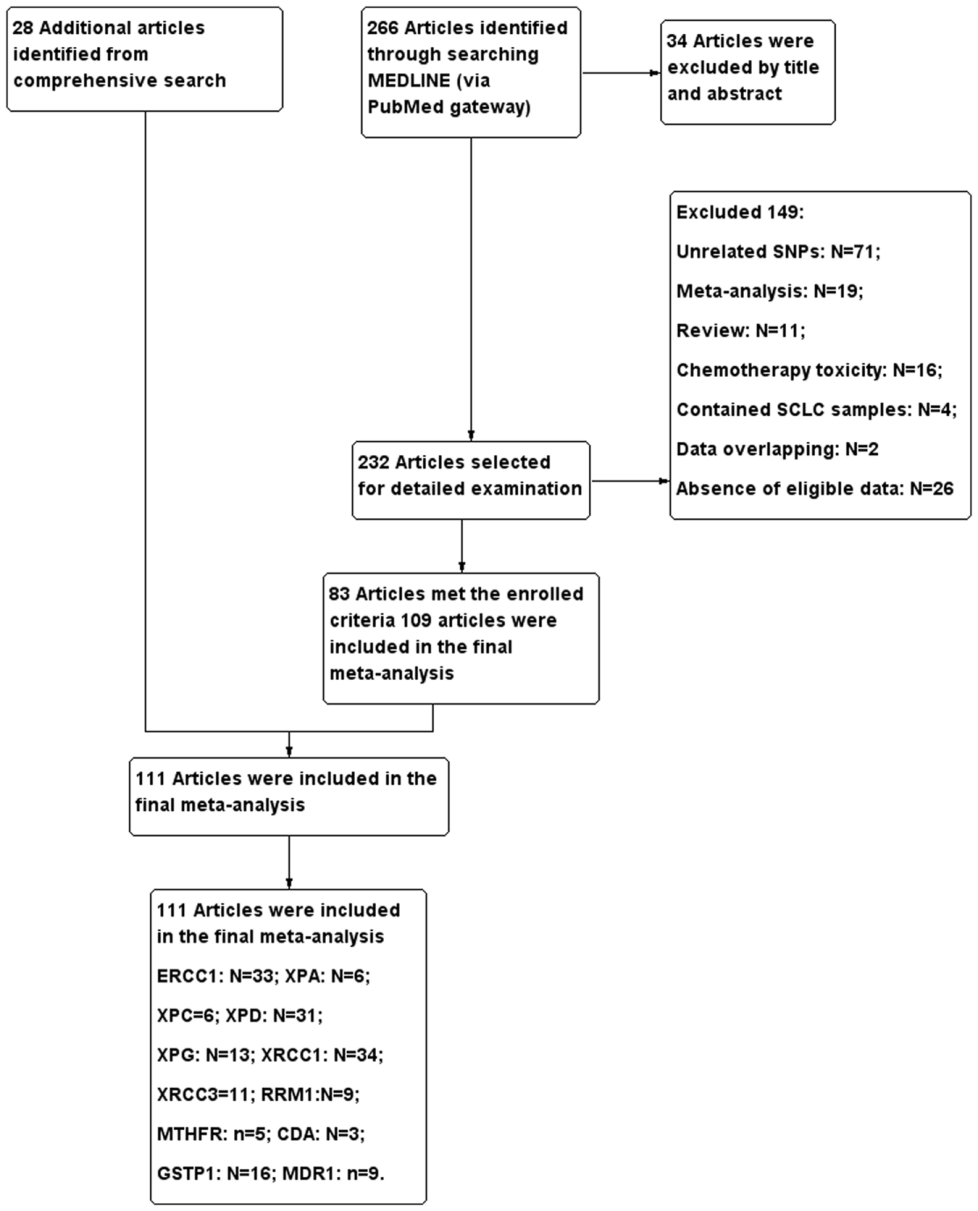
Flow diagram of the study selection process for the current meta-analysis.

After the process of selection, a total of 111 studies met the inclusion criteria and totally 18,196 NSCLC subjects (between the ages of 51 to 84) who accepted PBC were included in the final meta-analysis. More than 80% of these articles focused on the advanced NSCLC (in disease stages of III–IV). The process of selecting publications is presented in Fig. 1 and more details about the characteristics of the studies included are listed in Table 1.

**Table 1:** The baseline characteristics of the studies included in this meta-analysis.

First author (Year)	Ethnicity (country)	Sample size	Male/female	Median age	Disease stage	Chemotherapeutic drugs	Outcomes	Genotyping method	SNPs	Ref.
Camps, C. (2003)	Caucasian (Spain)	39	34/5	64 (27–82)	IIIB-IV	DDP+GEM	OR	Direct sequencing	*XPD* rs1799793 rs13181	12
Ryu, J. S. (2004)	Asian (Korea)	109	88/21	60 (32–78)	IIIB-IV	DDP+TAX/GEM/DOC	OR	SNaPShot assay	*ERCC1* rs11615 *XPD* rs1799793 rs13181	13
Gurubhagavatula, S. (2004)	Caucasian (USA)	103	53/50	58 (32–77)	IIIA-IV	DDP/CBP-based	OS	PCR-RFLP	*XPD* rs1799793 *XRCC1* rs25487	14
Isla, D. (2004)	Caucasian (Span)	62	48/14	62 (35–78)	IIIB-IV	DDP+DOC	OR	TaqMan	*ERCC1* rs11615, *XPD* rs13181 rs1799793, *RRM1* rs12806698, *MDR1* rs1045642	15
Zhou, W. (2004)	Caucasian (USA)	128	66/62	60 (32–78)	IIIA–IV	Platinum based	OS	PCR-RFLP	*ERCC1* rs11615 rs3212986	16
Wang, Z. H. (2004)	Asian (China)	105	59/46	56 (30–74)	IIIB–IV	DDP/CBP+NVB/TAX/DOC	OR	PCR-RFLP	*XECC1* rs1799782	17
Yuan, P. (2005)	Asian (China)	200	130/70	56 (30–74)	IIIB–IV	Platinum based	OR	PCR-RFLP	*ERCC1* rs3212986, *XPD* rs13181, *XPC* PAT	18
Lu, C. (2006)	Caucasian+Mexican/African American	425	236/198	NR	III–IV	Platinum based	OS	PCR-RFLP	*GSTP1* rs1695	19
de Las, P. R. (2006)	Caucasians (Span)	135	125,10	62 (31–81)	IIIB- IV	DDP+GEM	OS	TaqMan	*ERCC1* rs11615, *XPD* rs1799793, *XRCC1* rs25487	20
Booton, R. (2006)	Caucasian (UK)	108	74/34	62.5 (35–80)	III–IV	DDP/CBP-based	OR	PCR-RFLP Direct sequencing	*XPD* rs13181 rs1799793	21
Yuan, P. (2006)	Asian (China)	200	130/70	56 (30‑74)	IIIB- IV	DDP/CBP+NVB/TAX/DOC	OR	PCR‑RFLP	*XRCC1* rs1799782	22
Booton, R. (2006a)	Caucasian (UK)	108	74/34	62.5 (35–80)	III-IV	DDP/CBP-based	OR, OS	PCR-RFLP Direct sequencing	*GSTP1* rs1695	23
Shi, M. (2006)	Asian (China)	97	67/30	60 (22–81)	II-IV	Platinum based	OR	PCR-RFLP	*MTHFR* rs1801133	24
Shi, M. (2006a)	Asian (China)	112	81/31	60 (22–81)	II-IV	Platinum based	OR	PCR-RFLP	*XRCC1* rs25487 rs1799782	25
Su, D. (2007)	Asian (China)	76	179/51	58 (28–80)	IIIA–IV	Platinum based	OR	TaqMan	*ERCC1* rs11615	26
Sun, X. C. (2007)	Asian (China)	96	62/34	58 (34–77)	IV	DDP/CBP-based	OR	PCR-cDNA chip	*XPA* rs1800975	27
Song, D G. (2007)	Asian (China)	166	97/69	56 (30–68)	IIIB-IV	DDP+NVB/DOC/GEM	OR	PCR-RFLP	*XPD* rs1799793	28
Yu, Q Z. (2007)	Asian (China)	101	78/23	57 (30–72)	III-IV	DDP-based	OR	PCR-RFLP	*XPG* rs17655, *MDR1* rs1045642	29
Pan, J. H. (2008)	Asian (China)	69	48/21	55 (30–76)	IIIB-IV	DDP+NVP	OR	PCR-RFLP	*MDR1* rs1045642	30
Tibaldi, C. (2008)	Caucasian (Italy)	65	51/14	65 (44–77)	IIIB–IV	DDP+GEM	OR, OS	TaqMan	*ERCC1* rs11615, *XPD* rs13181 rs1799793, *CDA* rs2072671	31
Wu, X. (2008)	Caucasian (USA)	229	135/94	NR	IIIB–IV	Cisplatin-based	OS	TaqMan	*ERCC1* rs3212986, *XPG* rs17655, *GSTP1* rs1695, *MDR1* rs1045642, *XPA* rs1800975, *XPC* rs2228001, *XPC* rs2228000	32
Din, Z H. (2008)	Asian (China)	116	85/31	60 (22–81)	IIB–IV	DDP+GEM	OR	PCR-RFLP	*XPD* rs13181	33
Liu, X Z. (2008)	Asian (China)	53	38/15	61 (28–74)	I-IV	DDP/CBP-based	OS	TaqMan	*XPD* rs13181,	34
Pan, J. H. (2009)	Asian (China)	54	38/16	55 (30–76)	IIIB-IV	DDP+DOC	OR	PCR-RFLP	*MDR1* rs1045642	35
Sun, X. (2009)	Asian (China)	82	53/29	59 (34–79)	IV	DDP/CBP-based	OR	3D DNA microarray genotyping	*XPG* rs1047768 rs17655 *XRCC1* rs25487 rs1799782	36
Feng, J. F. (2009)	Asian (China)	214	158/56	59 (21–75)	IIB-IV	Platinum-based	OR	PCR-RFLP	*RRM1* rs12806698	37
Feng, J. F. (2009a)	Asian (China)	115	78/37	59.6 (34–84)	III–IV	DDP/CBP-based	OR	DNA microarray genotyping	*XPA* rs1800975	38
Kalikaki, A. (2009)	Caucasian (Greece)	119	101/18	61 (39–85)	IIIA-IV	Platinum-based	OR, OS	PCR-RFLP Direct sequencing	*ERCC1* rs3212986, *XPD* rs13181 rs1799793, *GSTP1* rs1695	39
Hong, C. Y. (2009)	Asian (China)	164	99/65	61 (27–84)	IIIB–IV	DDP+NVP	OR	PCR-RFLP	*XRCC1* rs25487 rs1799782	40
Gao, C M. (2009)	Asian (China)	57	44/13	59 (38–77)	II–IV	DDP+GEM	OR	PCR-RFLP	*XRCC1* rs1799782	41
Hu, S N. (2009)	Asian (China)	214	158/56	59 (22–81)	II–IV	Platinum based	OR	PCR-RFLP	*RRM1* rs12806698	42
Takenaka, T. (2010)	Asian (Japan)	122	75/47	69 (30–86)	I–III	platinum-based	OS	PCR-RFLP Direct sequencing	*ERCC1* rs11615 rs3212986	43
Sun, N. (2010)	Asian (China)	113	76/37	59.6 (34–84)	IIIA-IV	DDP/CBP-based	OR	3-D polyacrylamide gel-based DNA microarray	*GSTP1* rs1695	44
Chen, S. (2010)	Asian (China)	95	76/19	58 (35–77)	IIIB–IV	Platinum based	OR	LDR	*ERCC1* rs11615, *MDR1* rs1045642	45
Li, F. (2010)	Asian (China)	115	78/37	60 (NR)	IIIB-IV	platinum-based	OR	3-D polyacrylamide gel-based DNA microarray	*ERCC1* rs11615 rs3212986*XPD* rs13181	46
Zhou, C. (2010)	Asian (China)	130	74/56	61 (30–78)	IIIB-IV	DDP/CBP+NVB/TAX/GEM	OR	TaqMan	*ERCC1* rs11615, *XRCC3* rs861539	47
Zhu, X. L. (2010)	Asian (China)	96	64/32	57 (34–79)	III-IV	DDP/CBP-based	OR	DNA microarray genotyping	*XPC* rs2228001 rs2228000	48
Wang, J. (2010)	Asian (China)	90	63/27	55 (33–73)	III-IV	DDP+NVB/TAX/GEM/DOC	OR	Direct sequencing	*ERCC1* rs11615 rs3212986	49
Yuan, P. (2010)	Asian (China)	199	129/70	56 (29–74)	IIIA-IV	platinum-based	OS, PFS	PCR-RFLP	*XRCC1* rs25487 rs25489 rs1799782	50
Okuda, K. (2011)	Asian (Japan)	90	73/17	NR	I-IV	platinum-based	OS	PCR-RFLP	*ERCC1* rs11615 rs3212986	51
Vinolas, N. (2011)	Caucasian (Spain)	94	79/15	61 (37–77)	IIIB–IV	DDP+NVP	OR, OS	5′ nuclease allelic discrimination assay	*ERCC1* rs11615, *XPD* rs13181 rs1799793, *MDR1* rs1045642, *RRM1* rs12806698	52
Liu, L. (2011)	Asian (China)	199	129/70	56 (29–74)	IIIA-IV	Platinum-based	OS, PFS	PCR–RFLP	*XPD* rs13181	53
KimCurran, V. (2011)	Asian (China)	300	201/99	60 (33–78)	IIIB-IV	DDP/CBP+NVB/TAX/GEM	OR	RT-PCR	*ERCC1* rs3212986	54
Cui, L. H. (2011)	Asian (China)	101	62/39	58 (27–76)	IIIB-IV	DDP/CBP-based	OR	RT- PCR	*MTHFR* rs1801133	55
Ryu, J. S. (2011)	Asian (Korea)	298	236/62	63 (28–89)	IIIA-IV	DDP+GEM/TAX	OS	SBE	*RRM1* rs12806698	56
Zhou, F. (2011)	Asian (China)	111	67/44	57 (42–71)	IV	DDP/CBP+DOC/GEM/NVB/PEM	OR	Direct sequencing	*XRCC1* rs25487, *GSTP1* rs1695	57
Zhai, Y. N. (2011)	Asian (China)	163	98/65	61 (27–84)	IV	DDP+NVB	OR	PCR-RFLP	*XPC* rs2228001 rs2228000 PAT	27
Ludovini, V. (2011)	Caucasian (Italy)	192	142/50	63 (25–81)	IIIB-IV	DDP- based	OR	TaqMan	*ERCC1* rs11615 *XPD* rs13181, *XRCC3* rs861539	58
Xu, C. (2011)	Asian (China)	130	90/40	NR	IIIB-IV	Platinum-based	OR	PCR-RFLP	*XRCC1* rs25487 rs1799782, *XRCC3* rs861539	59
Yan, P. W. (2011)	Asian (China)	103	67/36	61 (39–79)	IIIB–IV	Platinum-based	OR	RT-PCR	*MDR1* rs1045642	60
Cheng, H. Y. (2011)	Asian (China)	120	82/38	58 (34–77)	NR	DDP/CBP-based	OR	Two-color fluorescent probe hybridization	*XRCC1* rs25487	61
Jia, X F. (2011)	Asian (China)	89	45/44	NR	III-IV	DDP/CBP+DOC/GEM	OR	Direct sequencing	*XPG* rs1047768, *XPA* rs1800975	62
Li, D R. (2011)	Asian (China)	89	64/25	59 (21–84)	IIIA-IV	DDP-based	OR	Direct sequencing	*XRCC1* rs25487	63
Li, D. R. (2011a)	Asian (China)	89	64/25	59 (21–84)	IIIA-IV	DDP-based	OR	Direct sequencing	*XPD* rs1799793	64
Zhao, W. (2011)	Asian (China)	151	92/59	62 (32–82)	IIIB-IV	DDP/CBP-based	OR	TaqMan	*XRCC1* rs25487	65
Zhou, F. (2011a)	Asian (China)	94	55/39	57 (42–71)	IIIB-IV	DDP-based	OR	Direct sequencing	*XRCC1* rs25487	66
Ren, S. (2012)	Asian (China)	340	232/108	60 (30–78)	IIIB-IV	DDP+NVB/GEM/TAX/DOC	OR, OS	TaqMan	*XPD* rs13181, *RRM1* rs12806698, *XRCC3* rs861539, *XPC* rs2228001 rs2228000	67
Dong, J. (2012)	Asian (China)	568	434/134	60 (25–83)	III–IV	Platinum based	OS	TaqMan	*ERCC1* rs11615, *XRCC1* rs25487, *XPC* rs2228000	68
Li, D. (2012)	Asian (China)	89	64/25	59 (21–84)	III-IV	DDP+NVB/TAX, DDP+GEM/DOC	OR	PCR-RFLP	*ERCC1* rs11615 *XPD* rs13181, *XRCC1* rs25487	69
Joerger, M. (2012)	Caucasian (Netherlands)	137	77/60	59.7 (37–79)	IIIB-IV	DDP+GEM	OR, OS, PFS	DNA sequencing	*ERCC1* rs11615, *XPD* rs1799793, *RRM1* rs12806698, *CDA* rs2072671, *XRCC3* rs861539	70
Cheng, J. (2012)	Asian (China)	142	89/53	62 (43–81)	IIIB-IV	DDP+NVB/TAX	OR	Direct sequencing	*ERCC1* rs11615	71
Li, W. (2012)	Asian (China)	217	148/69	59 (24–83)	NR	Platinum-based	OR	PCR-RFLP	*GSTP1* rs1695	72
Chen, X. (2012)	Asian (China)	355	248/107	60 (32–78)	IIIB-IV	DDP/CBP-based	OR	TaqMan	*XPD* rs13181, *XRCC3* rs861539	73
Wu, W. (2012)	Asian (China)	353	246/107	57 (32–80)	III- IV	DDP+NVB/TAX/GEM/DOC	OR, OS	Direct sequencing	*XPD* rs13181 rs1052555 rs238406	74
Butkiewicz, D. (2012)	Caucasian (Poland)	171	NR	NR	I–IV	Platinum based	OS, PFS	PCR-RFLP	*XPD* rs1799793l, *XRCC3* rs861539	75
Krawczyk, P. (2012)	Caucasian (Poland)	43	33/10	63 (NA)	IIIB–IV	Platinum based	OR	PCR-RFLP	*ERCC1* rs11615	76
Liao, W. Y. (2012)	Asian (Taiwan)	62	35/27	57 (36–78)	III- IV	DDP+GEM	OR, OS	TaqMan	*ERCC1* rs11615 rs3212986 *XRCC1* rs25487, *XRCC3* rs861539	77
Dogu, G. G. (2012)	Caucasian (Turkey)	79	72/7	60 (32–84)	IB-IV	Platinum based	OS	PCR-RFLP	*MDR1* rs1045642	78
Ke, H. G. (2012)	Asian (China)	460	334/126	55 (32–79)	I-IV	DDP-based	OS	PCR-CTPP	*XRCC1* rs25487 rs1799782, *GSTP1* rs1695, *XRCC3* rs861539	79
Lv, H Y. (2012)	Asian (China)	85	49/36	56 (36–71)	NR	DDP+DOC/GEM/NVB/MTA	OR	Direct sequencing	*XPG* rs1047768, *GSTP1* rs1695	80
Zhang, Y P. (2012)	Asian (China)	62	38/24	58 (37–72)	IIIB-IV	DDP+NVP/TAX/GEM	OR	TaqMan	*GSTP1* rs1695	81
Provencio, M. (2012)	Caucasian (Spain)	180	157/23	62 (39–78)	IIIB-IV	DDP+NVB	OR, PFS	TaqMan	*XRCC3 rs861539*	82
He, C. (2013)	Asian (China)	228	141/87	60 (19–84)	III-IV	DDP/CBP-based	OR	PCR-RFLP	*XPG* rs2296147	83
Hong, W. (2013)	Asian (China)	135	90/45	56 (25–72)	III-IV	DDP/CBP+GEM	OR	TaqMan	*ERCC1* rs11615 rs3212986, *MTHFR* rs1801133	84
Liu, H N. (2013)	Asian (China)	62	38/24	58 (37–72)	NR	DDP-based	OR	Taqman	*XRCC1* rs25487	85
Zhao, W. (2013)	Asian (China)	147	92/55	60 (32–82)	IIIB-IV	platinum-based	OR, OS, PFS	TaqMan	*XRCC1* rs25487 rs1799782	86
Li, X. D. (2013)	Asian (China)	496	324/172	63 (33–79)	IIIA-IV	platinum-based	OR, OS, PFS	PCR-SBE	*XPD* rs13181 rs1799793 rs1052555 rs238406,	87
Li, W. J. (2013)	Asian (China)	45	23/22	63 (39–81)	IIIB-IV	DDP+PEM	OR	Taqman	*MTHFR* rs1801133	88
Cheng, H. (2013)	Asian (China)	115	78/37	59.6 (34–84)	IIIB-IV	Platinum-based	OS, PFS	3-D polyacrylamide gel-based DNA	*XPD* rs13181, *XPA* rs1800975	89
Zhang, T. (2013)	Asian (China)	475	306/145	64 (32–76)	III-IV	DDP+DOC, DDP/CBP+GEM/NVB	OR, OS, PFS	TaqMan	*XPG* rs1047768 rs17655 rs2296147 rs873601	90
Lee, S. Y. (2013)	Asian (Korea)	382	311/71	NR	III-IV	DDP+TAX	OR, OS	Sequenome mass spectrometry-based	*XPD* rs1052555, *XRCC1* rs25487	91
Mlak, R. (2013)	Caucasian (Poland)	62	43/19	61 (38–76)	IIIA-IV	Platinum-based	OS	PCR-RFLP	*RRM1* rs12806698	92
Yuli, Y. (2013)	Asian (China)	433	284/149	61 (33–79)	IIIA-IV	DDP/CBP-based	OS, PFS	Taqman	*XPG* rs17655	93
Lu, H D. (2013)	Asian (China)	100	54/46	61 (41–82)	III-IV	DDP+NVB/TAX	OR	PCR-RFLP	*ERCC1* rs11615	94
Sheng, G F. (2013)	Asian (China)	62	38/24	58 (37–72)	NR	DDP-based	OR	Taqman	*XRCC1* rs25487	95
Yang, W J. (2013)	Asian (China)	54	38/16	56 (30–73)	III-IV	DDP/CBP-based	OR	PCR-RFLP	*XRCC1* rs1799782, *RRM1* rs12806698	96
Zhang, Y P. (2013)	Asian (China)	62	38/24	58 (37–72)	NR	DDP+NVB/TAX/GEM/PEM	OR	Direct sequencing	*XPD* rs13181	97
Zhou, G R. (2013)	Asian (China)	204	120/84	61 (45–75)	NR	DDP -based	OR	MALDI-TOF-MS	*XRCC1* rs25487	98
Huang, S. J. (2014)	Asian (China)	187	124/63	NR	IIIA-IV	Platinum-based	OR, OS	MALDI-TOF-MS	*ERCC1* rs11615 rs3212986, rs2298881	99
Zhang, L. (2014)	Asian (China)	375	249/126	NR	IIIA-IV	CBP+NVP+DDP, DDP+DOC	OR, OS, PFS	Sequenom MassARRAY platform	*XPD* rs13181 rs1799793 rs1052555 rs238406, *XRCC1* rs25487 rs1799782	100
Jin, Z. Y. (2014)	Asian (China)	378	297/81	62.4 (36–78)	I-IV	DDP+GEM/DOC/NVP/TAX	OR, OS	PCR-RFLP	*XPG* rs1047768 rs17655 *XRCC1* rs25489, *XRCC3* rs861539	101
Hu, W. (2014)	Asian (China)	277	184/93	63.1 (29–75)	IIIA-IV	Platinum-based	OS, PFS	PCR-RFLP	*XPG* rs1047768 rs17655 rs2296147 rs873601	102
Peng, Y. (2014)	Asian (China)	235	180/55	58 (29–84)	IIIA-IV	DDP+TAX/DOC/GEM	OR, OS	PCR-CTTP	*XRCC1* rs25487	103
Zhou, M. (2014)	Asian (China)	93	56/37	61.5 (NR)	IIIB-IV	DDP+GEM	OR	PCR-RFLP	*XPD* rs13181 rs1799793, *CDA* rs2072671	104
Zhao, X. (2014)	Asian (China)	192	132/60	60.8 (26–79)	IIIA-IV	Platinum-based	OR, OS	MALDI-TOF-MS	*ERCC1* rs3212986 rs11615 rs2298881	105
Lv, H. (2014)	Asian (China)	91	54/37	59 (34–80)	IIIB-IV	DDP+TAX/GEM/NVP	OR	TaqMan-MGB	*GSTP1* rs1695	106
Krawczyk, P. (2014)	Caucasian (Poland)	115	59/56	61 (NR)	II-IV	DDP/CBP+PEM	OS	HRM, PCR-RFLP	*ERCC1* rs11615	107
Sullivan, I. (2014)	Caucasian (Spain)	161	125/36	63.7 (36–85)	IIIA-IV	DDP/CBP-based	OR, OS	Dynamic array chips	*ERCC1* rs3212986 rs11615, *XPD* rs13181 rs1799793, *XPG* rs1047768 rs17655, *XRCC1* rs25487 rs1799782, rs25489, *XPA* rs1800975	108
Dong, C M. (2014)	Asian (China)	92	38/54	57 (40–6)	IIIB-IV	Platinum-based	OR	PCR-RFLP	*MTHFR* rs1801133	109
Liu, D. (2014)	Asian (China)	378	297/81	62.4 (36–78)	I-IV	DDP+GEM/DOC/NVP/TAX	OR, OS	PCR-RFLP	*XPG* rs1047768 rs17655 *XRCC1* rs25487 rs1799782	110
Kou, G. (2014)	Asian (China)	50	14/36	56 (45–78)	IIIB-IV	DDP+NVP	OR	PCR-RFLP	*ERCC1* rs3212986	111
Kalikaki, A. (2015)	Caucasian (Greece)	107	90/17	60 (37–78)	IIIB-IV	DDP/CBP-based	OR, OS, PFS	PCR-RFLP	*ERCC1* rs3212986, *XRCC1* rs25487	112
Zou, H. Z. (2015)	Asian (China)	246	170/76	64.3 (32–76)	IIIA-IV	DDP/CBP-based	OS, PFS	PCR-RFLP	*XPG* rs2296147 rs873601	113
Yuan, Z. J. (2015)	Asian (China)	47	42/5	59 (29–74)	III-IV	DDP+GEM	OR	DNA sequencing	*GSTP1* rs1695	114
Deng, J. H. (2015)	Asian (China)	97	66/31	57 (31–79)	IIIB-IV	DDP+GEM/NVP/TAX/DOC	OR, PFS	DNA pyrosequencing	*XRCC1* rs25487, *GSTP1* rs1695	115
Shi, Z. H. (2015)	Asian (China)	240	155/85	61.5 (34–78)	III-IV	DDP+GEM/NVP/TAX/DOC	OR, OS	PCR-RFLP	*ERCC1* rs11615 rs3212986 rs2298881	116
Han, B. (2015)	Asian (China)	325	116/209	NR	IIIB-IV	DDP+GEM/NVP/TAX/DOC	OR, OS	PCR-RFLP	*XRCC1* rs25487 rs1799782 rs25489, *GSTP1* rs1695	117
Li, P. (2015)	Asian (China)	142	89/53	62 (43–81)	IIIB-IV	DDP+NVP	OR	PCR-RFLP	*XPD* rs13181 rs1799793	118
Liu, J. Y. (2015)	Asian (China)	322	226/140	62.5 (37–81)	IIIB-IV	DDP+GEM/NVP/TAX/DOC	OR, OS	PCR-RFLP	*XRCC1* rs25487 rs1799782, *GSTP1* rs1695	119
Wu, G. (2015)	Asian (China)	282	181/101	NR	IIIA-IV	DDP-based	OR, OS	PCR-RFLP	*GSTP1* rs1695	120
Zhu, M Z. (2015)	Asian (China)	68	40/28	NR	IIIB-IV	DDP/CBP-based	OR	PCR-RFLP	*ERCC1* rs11615	121

NR, no report; DDP, cisplatin; CBP, carboplatin; GEM, gemcitabine; NVP, vinorelbine; PEM, pemetrexed; TAX, taxol/paclitaxel; DOC, docetaxel; LDR, Ligase detection reactions; PCR-RFLP, polymerase chain reaction-restriction fragment length polymorphism; SBE, single base extension; HRM, High Resolution Melt; MALDI-TOF-MS, matrix-assisted laser desorption/ionization time-of flight mass.

### Meta-analysis findings

#### Genetic variants associated with response to platinum drugs

As shown in Table 2, we conducted 74 primary meta-analyses and 64 subgroup meta-analyses sorted by ethnicity to study the associations between 24 SNPs of 12 genes and the responses to PBC in NSCLC patients. Of the 138 performed meta-analyses, 26 (19%) resulted in statistically significant (*P* < 0.05), with the remaining 112 being non-significant. For ORR, RR < 1 indicated that patients carrying the allele or genotype had a disadvantageous response, RR > 1 donated that the allele carriers had a favorable response. Pooled RR with 95% CI of individual SNPs identified as statistically associated with favorable responses to PBC were listed as follows: *XRCC1* rs25487 (AA vs. GG: overall RR = 1.27, 95% CI = 1.02–1.58), *XRCC1* rs1799782 (CT vs. CC: overall RR = 1.22, 95% CI = 1.03–1.44; TT vs. CC: overall RR = 1.29, 95% CI = 1.07–1.56; CT+TT vs. CC: overall RR = 1.22, 95% CI = 1.04–1.42), *XRCC3* rs861539 (CT VS CC: Caucasian RR = 1.46, 95% CI = 1.06–1.99 and overall RR = 1.31, 95% CI = 1.07–1.59; TT VS CC: Caucasian RR = 1.59, 95% CI = 1.07–2.36 and overall RR = 1.48, 95% CI = 1.12–1.97; TT+CT VS CC: Caucasian RR = 1.48, 95% CI = 1.10–2.01 and overall RR = 1.28, 95% CI = 1.07–1.52), *XPA* rs1800975 (AG VS AA: Asian RR = 2.17, 95% CI = 1.29–3.64 and overall RR = 1.74, 95% CI = 1.18–2.57), *GSTP1* rs1695 (GG vs. AA: overall RR = 1.45, 95% CI = 1.20–1.74; AG+GG vs. AA: Asian RR = 1.47, 95% CI = 1.11–1.95 and overall RR = 1.37, 95% CI = 1.06–1.76). Pooled RR with 95% CI of individual SNPs identified as statistically associated with unfavorable responses were presented below: *ERCC1* rs3212986 (AA vs. CC: Asian RR = 0.71, 95% CI = 0.54–0.94 and overall RR = 0.72, 95% CI = 0.56–0.94), *XPD* rs13181 (CA+CC vs. AA: Asian RR = 0.83, 95% CI = 0.71–0.98), *XPD* rs1799793 (AA vs. GG: Asian RR = 0.20, 95% CI = 0.05–0.76), *MTHFR* rs1801133 (CT vs. CC: mixed RR = 0.63, 95% CI = 0.44–0.89), *MDR1* rs1045642 (CT vs. CC: Asian RR = 0.69, 95% CI = 0.50–0.95 and overall RR = 0.73, 95% CI = 0.56–0.94; TT vs. CC: Asian RR = 0.47, 95% CI = 0.26–0.85 and overall RR = 0.52, 95% CI = 0.34–0.81; CT+TT vs. CC: Asian RR = 0.61, 95% CI = 0.48–0.79 and overall RR = 0.64, 95% CI = 0.52–0.80).

**Table 2:** The association between candidate gene polymorphisms and objective response.

Genetic model	Subgroup	No. of Study	Effect model	Pooled RR (95%CI)	I^2^ (%)	P_het_	Begg’s test (P-value)	Egger’s test (P-value)
*ERCC1* rs3212986								
AA VS CC	Asian	7	Fixed	0.71 (0.54,0.94)	29.2	0.206		
	Caucasian	1	Fixed	0.85 (0.47,1.53)	—	—		
	Overall	8	Fixed	0.72 (0.56,0.94)	18.7	0.282	0.458	0.115
CA VS CC	Asian	7	Fixed	0.91 (0.78,1.05)	46.3	0.083		
	Caucasian	1	Fixed	1.03 (0.80,1.31)	—	—		
	Overall	8	Fixed	0.92 (0.80,1.05)	41.3	0.103	0.322	0.259
CA+AA VS CC	Asian	10	Random	0.85 (0.68,1.05)	58.1	0.011		
	Caucasian	4	Random	1.19 (0.93,1.51)	25.1	0.261		
	Overall	14	Random	0.95 (0.80,1.13)	55.9	0.006	0.447	0.441
*ERCC1* rs11615								
CT VS CC	Asian	10	Random	0.87 (0.71,1.08)	50.9	0.032		
	Caucasian	6	Random	0.87 (0.60,1.26)	34.7	0.176		
	Overall	16	Random	0.87 (0.73,1.04)	41.8	0.040	0.528	0.823
TT VS CC	Asian	10	Random	1.04 (0.64,1.69)	76.8	0.000		
	Caucasian	6	Random	0.79 (0.57,1.10)	0.0	0.522		
	Overall	16	Random	0.96 (0.68,1.34)	66.7	0.000	1.000	0.475
CT+TT VS CC	Asian	17	Random	0.83 (0.68,1.02)	61.3	0.000		
	Caucasian	8	Random	0.97 (0.72,1.31)	38.5	0.123		
	Overall	25	Random	0.87 (0.74,1.03)	55.0	0.001	0.815	0.753
*ERCC1* rs2298881								
CA VS AA	Overall	3	Fixed	0.96 (0.79,1.15)	0.0	0.637	0.602	0.234
CC VS AA	Overall	3	Fixed	0.93 (0.70,1.24)	35.2	0.214	0.117	0.210
CA+CC VS AA	Overall	3	Fixed	0.95 (0.80,1.13)	16.5	0.302	0.602	0.364
XPA rs1800975								
AG VS AA	Asian	2	Random	2.17 (1.29,3.64)	79.6	0.027		
	Caucasian	1	Random	1.01 (0.61,1.68)				
	Overall	3	Random	1.74 (1.18,2.57)	77.8	0.011	0.117	0.156
GG VS AA	Asian	2	Random	1.09 (0.59,2.02)	85.3	0.009		
	Caucasian	1	Random	1.22 (0.75,1.99)				
	Overall	3	Random	1.14 (0.74,1.75)	71.2	0.031	0.602	0.175
AG+GG VS AA	Asian	3	Random	1.05 (0.72,1.52)	83.8	0.002		
	Caucasian	1	Random	1.11 (0.68,1.80)				
	Overall	4	Random	1.06 (0.77,1.45)	76.0	0.006	0.174	0.087
XPC rs2228000								
CT VS CC	Asian	3	Fixed	1.09 (0.84,1.41)	50.6	0.132	0.602	0.850
TT VS CC	Asian	3	Fixed	1.05 (0.71,1.56)	29.1	0.244	0.602	0.989
CT+TT VS CC	Asian	3	Fixed	1.09 (086,1.40)	37.0	0.204	0.117	0.030^b^
XPC rs2228001								
AC VS AA	Asian	2	Random	0.85 (0.58,1.25)	88.8	0.003		
CC VS AA	Asian	2	Random	0.83 (0.46,1.51)	56.1	0.131		
CC+AC VS AA	Asian	3	Random	0.90 (0.71,1.14)	79.1	0.008	0.602	0.065
XPC intron9 PAT								
SL VS SS	Asian	2	Fixed	0.93 (0.61,1.40)	0.0	0.322		
LL VS SS	Asian	2	Random	1.07 (0.29,3.94)	81.5	0.020		
SL+LL VS SS	Asian	2	Random	0.87 (0.38,1.89)	70.7	0.065		
*XPD* rs13181								
AC VS AA	Asian	8	Fixed	0.82 (0.65,1.04)	9.80	0.354		
	Caucasian	8	Fixed	1.04 (0.87,1.23)	0.0	0.935		
	Overall	16	Fixed	0.94 (0.81,1.08)	0.0	0.662	0.589	0.299
CC VS AA	Asian	2	Random	1.14 (0.09,14.34)	73.6	0.051		
	Caucasian	8	Random	1.09 (0.87,1.36)	0.0	0.584		
	Overall	10	Random	1.15 (0.88,1.51)	26.9	0.196	0.128	0.133
CA+CC VS AA	Asian	11	Fixed	0.83 (0.71,0.98)	0.0	0.580		
	Caucasian	9	Fixed	1.05 (0.90,1.24)	0.0	0.863		
	Overall	20	Fixed	0.92 (0.82,1.03)	0.0	0.615	1.000	0.414
*XPD* rs1799793								
AA VS GG	Asian	1	Random	0.20 (0.05,0.76)	—	—		
	Caucasian	8	Random	1.21 (0.96,1.51)	0.0	0.551		
	Overall	9	Random	1.03 (0.69,1.54)	52.6	0.031	0.144	0.247
GA VS GG	Asian	4	Random	0.88 (0.45,1.74)	74.6	0.008		
	Caucasian	9	Random	1.04 (0.87,1.24)	0.0	0.647		
	Overall	13	Random	0.99 (0.81,1.23)	35.3	0.100	0.625	0.969
GA+AA VS GG	Asian	6	Random	0.83 (0.59,1.17)	67.3	0.009		
	Caucasian	10	Random	1.04 (0.89,1.21)	0.0	0.746		
	Overall	16	Random	0.94 (0.79,1.11)	40.8	0.046	0.589	0.656
*XPD* rs1052555								
CT+TT VS CC	Overall	4	Random	0.92 (0.65,1.31)	67.5	0.026	1.000	0.813
*XPD* rs238406								
CA+AA VS CC	Overall	3	Fixed	0.96 (0.81,1.15)	0.0	0.667	0.117	0.007^b^
*XPG* rs1047768								
CT VS CC	Asian	3	Fixed	0.97 (0.79,1.20)	18.8	0.292		
	Caucasian	2	Fixed	1.17 (0.88,1.55)	0.0	0.777		
	Overall	5	Fixed	1.01 (0.85,1.21)	0.0	0.466	0.624	0.767
TT VS CC	Asian	3	Random	0.70 (0.27,1.81)	87.9	0.000		
	Caucasian	2	Random	0.92 (0.64,1.32)	0.0	0.735		
	Overall	5	Random	0.80 (0.49,1.32)	76.2	0.002	0.142	0.155
CT+TT VS CC	Asian	5	Random	0.86 (0.61,1.21)	68.3	0.013		
	Caucasian	2	Random	1.07 (0.84,1.37)	0.0	0.890		
	Overall	7	Random	0.94(0.75,1.19)	55.6	0.036	0.293	0.319
*XPG* rs17655								
CG VS CC	Asian	6	Fixed	1.09 (0.92,1.27)	22.6	0.264		
	Caucasian	1	Fixed	1.00 (0.58,1.72)	—	—		
	Overall	7	Fixed	1.08 (0.93,1.26)	8.2	0.366	0.453	0.230
GG VS CC	Asian	6	Fixed	1.20 (0.99,1.45)	20.1	0.282		
	Caucasian	1	Fixed	1.16 (0.71,1.88)	—	—		
	Overall	7	Fixed	1.19 (0.99,1.43)	4.5	0.392	0.652	0.417
CG+GG VS CC	Asian	6	Fixed	1.12 (0.97,1.29)	38.1	0.152		
	Caucasian	1	Fixed	1.11 (0.68,1.80)	—	—		
	Overall	7	Fixed	1.12 (0.97,1.29)	25.7	0.233	0.652	0.495
*XPG* rs2296147								
CT VS CC	Overall	2	Fixed	1.14 (0.84,1.54)	0.0	0.477		
TT VS CC	Overall	2	Fixed	1.34 (0.92,1.97)	0.0	0.547		
CT+TT VS CC	Overall	2	Fixed	1.22 (0.96,1.56)	0.0	0.863		
*XRCC1* rs25487								
GA VS GG	Overall	15	Random	1.08 (0.94,1.24)	60.8	0.001	0.458	0.375
AA VS GG	Overall	15	Random	1.27 (1.02,1.58)	66.7	0.000	0.216	0.095
GA+AA VS GG	Overall	23	Random	0.89 (0.76,1.05)	78.5	0.000	0.013^a^	0.004^b^
*XRCC1* rs1799782								
CT VS CC	Overall	13	Random	1.22 (1.03,1.44)	63.4	0.001	0.051	0.032^b^
TT VS CC	Overall	13	Random	1.29 (1.07,1.56)	50.5	0.019	1.000	0.735
CT+TT VS CC	Overall	14	Random	1.22 (1.04,1.42)	65.1	0.000	0.139	0.082
*XRCC1* rs25489								
GA VS GG	Overall	2	Fixed	0.99 (0.81,1.22)	0.0	0.801		
AA VS GG	Overall	2	Fixed	0.96 (0.76,1.22)	0.0	0.712		
XRCC3 rs861539								
CT VS CC	Asian	3	Fixed	1.20 (0.94,1.53)	0.0	0.588		
	Caucasian	3	Fixed	1.46 (1.06,1.99)	26.3	0.257		
	Overall	6	Fixed	1.31 (1.07,1.59)	0.0	0.502	0.005^a^	0.009^b^
TT VS CC	Asian	1	Fixed	1.36 (0.91,2.02)				
	Caucasian	3	Fixed	1.59 (1.07,2.36)	0.0	0.935		
	Overall	4	Fixed	1.48 (1.12,1.97)	0.0	0.921	0.04^a^	0.001^b^
TT+CT VS CC	Asian	5	Fixed	1.16 (0.94,1.44)	0.0	0.764		
	Caucasian	3	Fixed	1.48 (1.10,2.01)	0.0	0.472		
	Overall	8	Fixed	1.28 (1.07,1.52)	0.0	0.723	0.001^a^	0.000^b^
*RRM1* rs12806698								
AA VS CC	Overall	4	Fixed	0.61 (0.33,1.12)	0.0	0.927	0.734	0.434
CA VS CC	Overall	6	Fixed	1.02 (0.86,1.21)	0.0	0.944	1.000	0.765
CA+AA VS CC	Overall	6	Fixed	0.98 (0.83,1.16)	0.0	0.954	1.000	0.770
*MTHFR* rs1801133								
CT VS CC	Overall	5	Fixed	0.63 (0.44,0.89)	41.0	0.148‘	0.327	0.297
TT VS CC	Overall	5	Random	0.81 (0.38,1.74)	64.0	0.025	0.327	0.392
CT + TT VS CC	Overall	5	Random	0.66 (0.37,1.18)	64.8	0.023	0.624	0.598
*GSTP1* rs1695								
AG VS AA	Asian	5	Random	1.19 (0.92,1.54)	73.8	0.004		
	Caucasian	2	Random	0.94 (0.62,1.44)	0.0	0.529		
	Overall	7	Random	1.14 (0.91,1.41)	63.1	0.012	0.881	0.891
GG VS AA	Asian	4	Random	1.17 (0.71,1.91)	78.5	0.001		
	Caucasian	2	Random	0.73 (0.28,1.90)	—	—		
	Overall	5	Fixed	1.45 (1.20,1.74)	0.0	0.416	1.000	0.654
AG+GG VS AA	Asian	11	Random	1.47 (1.11,1.95)	81.1	0.000		
	Caucasian	2	Random	0.90 (0.59,1.36)	0.0	0.713		
	Overall	13	Random	1.37 (1.06,1.76)	78.0	0.000	0.625	0.283
*MDR1* rs1045642								
CT VS CC	Asian	3	Fixed	0.69 (0.50,0.95)	0.0	0.495		
	Caucasian	2	Fixed	0.81 (0.52,1.26)	0.0	0.421		
	Overall	5	Fixed	0.73 (0.56,0.94)	0.0	0.678	0.624	0.610
TT VS CC	Asian	3	Fixed	0.47 (0.26,0.85)	27.4	0.252		
	Caucasian	2	Fixed	0.62 (0.32,1.17)	0.0	0.939		
	Overall	5	Fixed	0.52 (0.34,0.81)	0.0	0.621	0.142	0.226
CT+TT VS CC	Asian	5	Fixed	0.61 (0.48,0.79)	0.0	0.590		
	Caucasian	2	Fixed	0.75 (0.49,1.14)	0.0	0.551		
	Overall	7	Fixed	0.64 (0.52,0.80)	0.0	0.722	0.652	0.739
CDA rs2072671								
AC VS AA	Asian	1	Fixed	1.48 (0.78,2.81)				
	Caucasian	2	Fixed	0.85 (0.56,1.30)	43.7	0.183		
	Overall	3	Fixed	0.99 (0.70,1.40)	48.6	0.143	0.602	0.829
CC VS AA	Caucasian	2	Random	0.62 (0.10,3.96)	70.8	0.065		
AC+CC VS AA	Asian	1	Random	1,48 (0.78,2.81)	70.6	0.064		
	Caucasian	2	Random	0.77 (0.36,1.64)				
	Overall	3	Random	0.95 (0.53,1.71)	65.6	0.055	0.602	0.802

^a^Begg’s test *P* < 0.05; ^b^Egger’s test *P* < 0.05.

#### Genetic variants associated with OS and PFS

Statistically significant results with HR > 1 indicated that patients carrying the allele or genotype harbored a poorer OS or PFS, while with HR < 1 meant better OS or PFS of patients. As for OS (Table 3), 52 meta-analyses were preformed to examine the influence of 22 SNPs in 11 genes on the overall survival. Seven results were identified as statistically significantly associated with OS. Of them, *ERCC1* rs11615 (CT+TT vs. CC: HR = 1.47, 95% CI = 1.15–1.88), *ERCC1* rs3212986 (AA vs. CC: HR = 2.06, 95% CI = 1.19–3.57), *XPD* rs13181 (AC+CC vs. AA: HR = 1.24, 95% CI = 1.07–1.44), and *XPD* rs1052555 (CT+TT vs. CC: HR = 1.71, 95% CI = 1.31–2.23) might be related to a poorer OS, while *XPG* rs873601 (GG vs. AA: HR = 0.67, 95% CI = 0.46–0.97), *XPG* rs2296147 (TT vs. CC: HR = 0.40, 95% CI = 0.27–0.61), and *XPD* rs1799793 (GA vs. GG: HR = 0.78, 95% CI = 0.62–0.99) might be potentially related to a better OS. No significant association was identified in the remaining SNPs. As for PFS (Table 4), 19 meta-analyses were conducted and 11 SNPs of 4 genes were investigated to explore their associations with the PFS of NSCLL patients. Our findings showed that patients with C allele of *XPD* rs13181 had a poorer PFS (AC+CC vs. AA: HR = 1.38, 95% CI = 1.10–1.73), and the T allele of *XPD* rs1052555 also indicated a poorer PFS (CT+TT vs. CC: HR = 1.97, 95% CI = 1.38–2.83).

**Table 3:** The association between candidate gene polymorphisms and OS.

Genetic model	No. of Study	Effect model	Pooled HR (95%CI)	I2%	P_het_	Begg’s test (P-value)	Egger’s test (P-value)
*ERCC1* rs3212986							
AA VS CC	4	Fixed	2.06 (1.19,3.57)	49.9	0.112	0.174	0.270
CA VS CC	5	Fixed	1.16 (0.83,1.63)	16.5	0.310	0.327	0.622
CA+AA VS CC	6	Random	0.97 (0.63,1.50)	81.1	0.000	0.851	0.356
*ERCC1* rs11615							
CT VS CC	6	Fixed	1.10 (0.89,1.37)	0.0	0.426	0.573	0.251
TT VS CC	8	Random	1.40 (0.92,2.16)	60.1	0.014	1.000	0.796
CT+TT VS CC	5	Fixed	1.47 (1.15,1.88)	0.0	0.682	0.624	0.597
*ERCC1* rs2298881							
AC VS AA	3	Fixed	1.20 (0.81,1.79)	0.0	0.526	0.602	0.644
CC VS AA	3	Fixed	1.20 (0.66,2.18)	0.0	0.437	0.117	0.151
XPA rs1800975							
AG+GG VS AA	2	Random	0.97 (0.73,1.29)	85.3	0.009		
XPC rs2228000							
CT VS CC	2	Random	0.74 (0.37,1.48)	85.5	0.009		
TT VS CC	2	Fixed	0.91 (0.56,1.50)	0	0.449		
CT+TT VS CC		Random	0.77 (0.40,1.48)	84.9	0.010		
XPC rs2228001							
CC+AC VS AA	2	Fixed	0.94 (0.74,1.20)	0.0	0.514		
*XPD* rs13181							
AC+CC VS AA	8	Fixed	1.24 (1.07,1.44)	7.70	0.371	0.458	0.645
*XPD* rs1799793							
AA VS GG	5	Random	1.09 (0.62,1.92)	65.3	0.021	0.624	0.595
GA VS GG	4	Fixed	0.78 (0.62,0.99)	0.0	0.419	0.497	0.422
GA+AA VS GG	6	Random	1.29 (0.94,1.76)	66.9	0.010	0.851	0.759
*XPD* rs1052555							
CT+TT VS CC	3	Fixed	1.71(1.31,2.23)	0.0.0	0.816		
*XPD* rs238406							
CA+AA VS CC	2	Fixed	1.26 (0.95,1.68)	0.0	0.913		
*XPG* rs1047768							
CT VS CC	2	Random	1.11(0.69,1.79)	59.3	0.117		
TT VS CC	3	Random	1.11 (0.45,2.78)	89.9	0.00	0.602	0.326
*XPG* rs17655							
CG VS CC	2	Fixed	0.98 (0.73,1.32)	0.0	0.743		
GG VS CC	2	Fixed	1.02 (0.68,1.51)	0.0	0.394		
CG+GG VS CC	2	Fixed	0.86 (0.68,1.08)	19.4	0.265		
*XPG* rs2296147							
CT VS CC	3	Fixed	0.79 (0.59,1.05)	0.0	0.920	0.602	0.376
TT VS CC	3	Fixed	0.40(0.27,0.61)	13.3	0.315	0.117	0.333
*XPG* rs873601							
AG VS AA	3	Fixed	0.91 (0.69,1.21)	0.0	0.548	1.000	0.878
GG VS AA	3	Fixed	0.67 (0.46,0.97)	0.5	0.366	0.602	0.710
*XRCC1* rs25487							
GA VS GG	13	Random	0.87 (0.71,1.07)	70.3	0.000	0.038^a^	0.029^b^
AA VS GG	11	Random	0.84 (0.52,1.36)	80.1	0.000	0.186	0.183
GA+AA VS GG	6	Random	0.96(0.68,1.36)	68.8	0.007	0.039^a^	0.019^b^
*XRCC1* rs1799782							
CT VS CC	7	Fixed	0.91 (0.76,1.08)	0.0	0.784	0.362	0.233
TT VS CC	7	Fixed	0.81 (0.63,1.04)	0.0	0.424	0.453	0.685
*XRCC1* rs25489							
GA VS GG	2	Fixed	0.85 (0.63,1.15)	41.3	0.192		
AA VS GG	2	Fixed	1.31 (0.65,2.65)	22.6	0.256		
XRCC3 rs861539							
CT VS CC	3	Fixed	0.95 (0.76,1.17)	0.0	0.630	0.117	0.064
TT VS CC	3	Fixed	1.01 (0.72,1.41)	46.1	0.156	0.602	0.935
TT+CT VS CC	2	Fixed	0.83 (0.61,1.13)	0.0	0.661		
*RRM1* rs12806698							
AA VS CC	2	Fixed	0.86 (0.47,1.58)	0.0	0.977		
AC VS CC	2	Fixed	0.91 (0.66,1.24)	0.0	0.513		
AC+AA VS CC	4	Random	1.01 (0.71,1.42)	66.7	0.029	0.174	0.391
*GSTP1* rs1695							
AG VS AA	8	Random	1.03 (0.82,1.28)	52.9	0.038	0.383	0.113
GG VS AA	5	Random	0.87(0.51,1.47)	71.2	0.008	0.624	0.535
AG+GG VS AA	2	Fixed	1.19 (0.92,1.55)	0.0	0.538		
*MDR1* rs1045642							
CT VS CC	3	Fixed	0.91 (0.66,1.25)	38.5	0.196	0.602	0.366
TT VS CC	3	Fixed	0.91 (0.64,1.29)	0.0	0.883	0.117	0.173
CDA rs2072671							
AC VS AA	2	Fixed	0.90 (0.63,1.29)	0.0	0.334		
CC VS AA	2	Random	1.80 (0.47,6.87)	80.6	0.023		

^a^Begg’s test *P* < 0.05; ^b^Egger’s test *P* < 0.05.

**Table 4:** The association between candidate gene polymorphisms and PFS.

Genetic model	No. of Study	Effect model	Pooled HR (95%CI)	I2%	P_het_	Begg’s test (P-value)	Egger’s test (P-value)
*XRCC1* rs25487							
GA VS GG	3	Fixed	0.91 (0.71,1.17)	0.0	0.376	0.602	0.273
AA VS GG	3	Fixed	0.72 (0.48,1.08)	29.2	0.243	0.602	0.571
GA+AA VS GG	5	Fixed	0.86 (0.72,1.05)	0.00	0.774	0.050	0.008^b^
*XRCC1* rs1799782							
CT VS CC	3	Fixed	1.06 (0.82,1.36)	0.0	0.777	0.117	0.461
TT VS CC	3	Fixed	1.00 (0.67,1.50)	8.8	0.334	0.117	0.429
CT+TT VS CC	3	Fixed	1.05 (0.83,1.34)	0.0	0.641	0.117	0.401
XRCC3 rs 86153							
CT VS CC	3	Fixed	0.86 (0.70,1.06)	0.0	0.895	0.221	0.562
**TT VS CC**	3	Fixed	0.94 (0.66,1.33)	0.0	0.372	0.117	0.166
*XPD* rs13181							
AC+CC VS AA	4	Fixed	1.38 (1.10,1.73)	0.0	0.965	0.042^a^	0.029^b^
*XPD* rs1799793							
GA+AA VS GG	4	Fixed	1.07 (0.86,1.33)	0.0	0.658	0.042^a^	0.013^b^
*XPD* rs1052555							
CT+TT VS CC	2	Fixed	1.97 (1.38,2.83)	0.0	0.815		
*XPD* rs238406							
CA+AA VS CC	2	Fixed	1.27 (0.89,1.81)	0.0	0.864		
*XPG* rs1047768							
CT VS CC	2	Fixed	1.08 (0.79,1.48)	17.7	0.270		
*XPG*rs17655							
CG VS CC	3	Fixed	0.85 (0.65,1.12)	0.0	0.555	0.602	0.242
GG VS CC	3	Fixed	0.69 (0.48,0.99)	0.0	0.974	0.117	0.077
*XPG* rs2296147							
CT VS CC	3	Fixed	0.80 (0.60,1.08)	0.0	0.503	0.602	0.353
TT VS CC	3	Fixed	0.51 (0.33,0.78)	17.8	0.296	0.602	0.455
*XPG* rs873601							
AG VS AA	3	Fixed	0.84 (0.63,1.13)	0.0	0.876	0.602	0.678
GG VS AA	3	Fixed	0.62 (0.41,0.91)	0.0	0.802	0.602	0.992

^a^Begg’s test *P* < 0.05; ^b^Egger’s test *P* < 0.05.

#### Heterogeneity and publication bias

A total of 54% (n = 97) of meta-analyses showed no heterogeneity (I^2^: 0 to 25%) and 14% (n = 25) presented moderate heterogeneity (I^2^: 25 to 50%), and large heterogeneity even extreme heterogeneity existed in other meta-analyses. Sensitivity analysis and subgroup analysis were also applied to find the source of heterogeneity. The clinical heterogeneity such as disease stages, different chemotherapy regimens might be the major reason for the large or extreme heterogeneity.

We used *P* value for Egger’s test to evaluate the potential publication bias. Our results suggested that effects of *XPD* rs238406 (CA+AA vs. CC), *XRCC1* rs25487 (GA+AA vs. GG), *XRCC1* rs1799782 (CT vs. CC) and *XRCC3* rs861539 (CT vs. CC, TT vs. CC and TT+CT vs. CC) on the ORR had significant publication bias. There was also some publication bias in analysis of the effects of *XRCC1* rs25487 (GA vs. GG, GA+AA vs. GG) on the OS. Three meta-analyses showed bias in the association of certain SNPs with PFS, including *XPD* rs13181 (AC+CC vs. AA), *XPD* rs1799793 (GA+AA vs. GG) and *XRCC1* rs25487 (GA+AA vs. GG). More details were listed in Tables 2 and 3.

#### False positive report probability

False positive findings regarding associations between genetic variants and diseases lead to a confounding effect. Here we assessed the FPRP to determine whether our finding was noteworthy. As shown in Table 5, 23 out of 35 results had FPRP lower than 0.2, with the prior probability set as 0.1 and the cut-off FPRP value as 0.2. The details of significant associations characterized by assessing FPRP are reported in Table 5.

**Table 5:** FPRP values for the SNPs associated with the response, OS and PFS of NSCLC patients receiving platinum-based chemotherapy.

Genetic/SNP	Genetic model	Subgroup	No. of study	Pooled RR of ORR	(95% CI)	Reported P-value	Power	FPRP based on prior		
								0.1	0.01	0.001
*ERCC1* rs3212986	AA VS CC	Asian	7	0.71	(0.54,0.94)	0.017	0.670	0.184^#^	0.712	0.961
	AA VS CC	Overall	8	0.72	(0.56,0.94)	0.016	0.714	0.166^#^	0.686	0.957
XRCC3 rs861539	CT VS CC	Caucasian	3	1.46	(1.06,1.99)	0.017	0.568	0.208	0.743	0.967
	CT VS CC	Overall	6	1.31	(1.07,1.59)	0.006	0.915	0.058^#^	0.405	0.873
	TT VS CC	Caucasian	3	1.59	(1.07,2.36)	0.021	0.386	0.332	0.846	0.982
	TT VS CC	Overall	4	1.48	(1.12,1.97)	0.007	0.537	0.108^#^	0.571	0.931
	TT+CT VS CC	Caucasian	3	1.48	(1.10,2.01)	0.012	0.534	0.169^#^	0.691	0.958
	TT+CT VS CC	Overall	8	1.28	(1.07,1.52)	0.005	0.965	0.043^#^	0.333	0.835
XPA rs1800975	AG VS AA	Asian	2	2.17	(1.29,3.64)	0.003	0.081	0.270	0.803	0.976
	AG VS AA	Overall	3	1.74	(1.18,2.57)	0.005	0.228	0.175^#^	0.700	0.959
*XPD* rs13181	CA+CC VS AA	Asian	11	0.83	(0.71,0.98)	0.028	0.995	0.202	0.735	0.966
*XPD* rs1799793	AA VS GG	Asian	1	0.20	(0.05,0.76)	0.047	0.069	0.861	0.985	0.999
*XRCC1* rs25487	AA VS GG	Overall	15	1.27	(1.02,1.58)	0.032	0.932	0.236	0.772	0.972
*XRCC1* rs1799782	CT VS CC	Overall	13	1.22	(1.03,1.44)	0.019	0.993	0.145^#^	0.651	0.950
	TT VS CC	Overall	13	1.29	(1.07,1.56)	0.009	0.940	0.076^#^	0.476	0.902
	CT+TT VS CC	Overall	14	1.22	(1.04,1.42)	0.010	0.996	0.085^#^	0.505	0.911
*MTHFR* rs1801133	CT VS CC	Overall	5	0.63	(0.44,0.89)	0.009	0.374	0.174^#^	0.699	0.959
*GSTP1* rs1695	GG VS AA	Overall	5	1.45	(1.20,1.74)	0.000	0.642	0.001^#^	0.010^#^	0.092^#^
*GSTP1* rs1695	AG+GG VS AA	Asian	11	1.47	(1.11,1.95)	0.008	0.556	0.109^#^	0.573	0.931
		Overall	13	1.37	(1.06,1.76)	0.014	0.761	0.140^#^	0.642	0.948
*MDR1* rs1045642	CT VS CC	Asian	3	0.69	(0.50,0.95)	0.023	0.584	0.261	0.796	0.975
		Overall	5	0.73	(0.56,0.94)	0.015	0.759	0.148^#^	0.657	0.951
	TT VS CC	Asian	3	0.47	(0.26,0.85)	0.013	0.124	0.476	0.909	0.990
		Overall	5	0.52	(0.34,0.81)	0.004	0.136	0.202	0.736	0.966
	CT+TT VS CC	Asian	5	0.61	(0.48,0.79)	0.000	0.250	0.006^#^	0.066^#^	0.417
		Overall	7	0.64	(0.52,0.80)	0.000	0.360	0.002^#^	0.024^#^	0.197^#^
*ERCC1* rs11615	CT+TT VS CC	Overall	5	1.47	(1.15,1.88)	0.002	0.564	0.033^#^	0.274	0.792
*ERCC1* rs3212986	AA VS CC	Overall	4	2.06	(1.19,3.57)	0.010	0.129	0.411	0.885	0.987
*XPD* rs13181	AC+CC VS AA	Overall	8	1.24	(1.07,1.44)	0.005	0.994	0.042^#^	0.324	0.829
*XPD* rs1799793	GA VS GG	Overall	4	0.78	(0.62,0.99)	0.041	0.902	0.291	0.819	0.979
*XPD* rs1052555	CT+TT VS CC	Overall	3	1.71	(1.31,2.23)	0.000	0.167	0.004^#^	0.043^#^	0.310
*XPG* rs873601	GG VS AA	Overall	3	0.67	(0.46,0.97)	0.034	0.511	0.374	0.868	0.985
*XPG* rs2296147	TT VS CC	Overall	3	0.40	(0.27,0.61)	0.000	0.009	0.021^#^	0.189^#^	0.702
*XPD* rs13181	AC+CC VS AA	Overall	4	1.38	(1.10,1.73)	0.005	0.765	0.058^#^	0.403	0.872
*XPD* rs1052555	CT+TT VS CC	Overall	2	1.97	(1.38,2.83)	0.000	0.070	0.030^#^	0.256	0.776

^#^FPRP value <0.2.

High-quality significant associations that emerged from the current meta-analysis were discussed below.

#### Excision Repairs Cross-complementation Groups 1 (*ERCC1*)

Data showed that *ERCC1* rs3212986 (C8092A) variant was related to the treatment response to PBC, and A allele may have poorer response comparing with C allele in Asians (AA vs. CC: pooled OR = 0.71, 95% CI = 0.54–0.94). Only moderate between-study heterogeneity was observed (I^2^ = 29.2%), and with a low FPRP when prior probability level was set as 0.1, suggesting that A allele of *ERCC1* rs3212986 might be specifically linked to the poorer response in Asians.

*ERCC1* rs11615 (C354T) was associated with OS, and T allele carriers might have unfavorable OS with HR being 1.47 and corresponding 95% CI being 1.15–1.88, and with no heterogeneity and low FPRP when prior probability level was set as 0.1, but subgroup classification by ethnicity were not performed.

#### Xeroderma Pigmentosum Group D (*XPD*)

Only the dominant model was used to analyze the relation between *XPD* rs13181 (A2251C) mutation and OS due to insufficient raw data. We found that the variant C allele was remarkably associated with the adverse OS in overall NSCLC patients treated with PBC (AC+CC vs. AA: HR = 1.24, 95% CI = 1.07–1.44). There was no heterogeneity and publication bias in the meta-analysis, and FPRP was low with the prior probability level being 0.1. C allele was also related to poor PFS with low FPRP at the high prior probability levels (AC+CC vs. AA: HR = 1.38, 95% CI = 1.10–1.73). No heterogeneity with statistical significance was observed, but the *P* value for Egger’s test showed that there was some publication bias in the meta-analysis. These results indicated that C allele was a risk allele for the poor clinical prognosis of NSCLC patients.

For other SNPs (rs1052555, C2133T) of *XPD*, we found that T allele was a risk allele and might be significantly associated with unfavorable OS (CT+TT vs. CC: HR = 1.71, 95% CI = 1.31–2.23). In the beginning, we included 4 articles in the meta-analysis and found that extreme heterogeneity and publication bias existed. After sensitivity analysis, we removed one article that was identified as the major source of heterogeneity, then I^2^ reduced to zero and no bias was observed from these data. The report had low FRPR with the prior probability level being 0.1 or 0.01. T allele was also related to poor PFS, and pooled HR was 1.97 and the 95% CI ranged from 1.38 to 2.83, though the report had low FPRP at high prior probability levels and no heterogeneity was observed. Further investigation with a larger sample size is needed to confirm the association between rs1052555 variant and prognosis of NSCLC patients.

#### Xeroderma Pigmentosum Group G (*XPG*)

*XPG* rs2296147 (T242C) might be associated with NSCLC patients’ prognosis receiving platinum drugs. We found that T allele acted as a protective allele with the carriers having favorable OS (TT vs. CC: HR = 0.40, 95% CI = 0.27–0.61), no heterogeneity and publication bias was detected, and the FPRP was low both at the high (0.1) and intermediate (0.01) prior probability levels. The strength of association needs to be further studied because of the small sample size of current meta-analysis.

#### X-Ray Cross-Complementing Group 1 (*XRCC1*)

Three genetic models were used to analyze the association between *XRCC1* rs1799782 (C580T) polymorphisms and ORR, and results confirmed the positive response of patients carrying T allele to PBC with a low FPRP at the high (0.1) prior probability level, but large between-study heterogeneity existed in the three meta-analyses ((CT vs. CC: HR = 1.22, 95% CI = 1.03–1.44, I^2^: 63.4%); (TT vs. CC: HR = 1.29, 95% CI = 1.07–1.56, I^2^: 50.5%); (CT+TT vs. CC: HR = 1.22, 95% CI = 1.04–1.42, I^2^: 65.1%)).

#### X-Ray Cross-Complementing Group 3 (*XRCC3*)

Results from subgroup meta-analysis sorted by ethnicity showed that T allele of *XRCC1* rs861539 (C241T) was associated with the positive response of PBC treatment in Caucasian population, three genetic models had consistent results (CT VS CC: RR = 1.46, 95% CI = 1.06–1.99; TT VS CC: RR = 1.59, 95% CI = 1.07–2.36; TT+CC VS CC: RR = 1.48, 95% CI = 1.10–2.01), no heterogeneity has been found. Begg’s test and Egger’s test revealed that some publication bias existed in the meta-analysis. However, Lower FRPR values suggested that the findings were statistically significant. Genetic variant of *XRCC1* rs861539 was not associated with OS and PFS in the current meta-analysis.

#### Methylenetetrahydrofolate Reductase (*MTHFR*)

T allele of *MTHFR* rs1801133 (C665T) might be related to the negative response, the report had low FPRP at the high (0.1) prior probability level, with pooled HR = 0.63, 95% CI = 0.44–0.89, I^2^ = 41.0% when comparing CT and CC genotypes. The other genetic models including TT vs. CC and CT+TT vs. CC didn’t show statistical significance.

#### Glutathione S-transferase P1 (*GSTP1*)

For *GSTP1* rs1695 (A313G), two genetic models showed consistent results about the association of the SNP with response (GG vs. AA: HR = 1.45, 95% CI = 1.20–1.74; AG+GG vs. AA: HR = 1.37, 95% CI = 1.06–1.76), the same effects were also observed in the Asian group by subgroup analysis in model AG+GG vs. AA (HR = 1.47, 95% CI = 1.11–1.95). However, we did not find a significant association in model AG vs. AA, low frequency of G allele and an insufficient sample size might be a major reason for it. We further assessed the FPRP value, and data showed low FPRP with probability level being 0.1. These results suggested that the G allele might play a protective role in the response of platinum-based treatment.

#### Multidrug resistance 1 (*MDR1*)

There were statistically significant associations between *MDR1* rs1045642 (T3435C) polymorphism and treatment response in both overall and Asian groups in three comparison genetic models (CT vs. CC, TT vs. CC, CT+TT vs. CC), and results are presented in Table 2. Three statistically significant findings with low FPRP were considered as noteworthy (CT vs. CC: overall RR = 0.73, 95% CI = 0.56–0.94; CT+TT vs. CC: Asian RR = 0.61, 95% CI = 0.48–0.79; CT+TT vs. CC: overall RR = 0.64, 95% CI = 0.52–0.80). Significant between-study heterogeneity and potential bias were not observed in all comparison models.

#### Biological pathways associated with platinum drugs treatment outcomes in NSCLC patients

**Figure 2:**
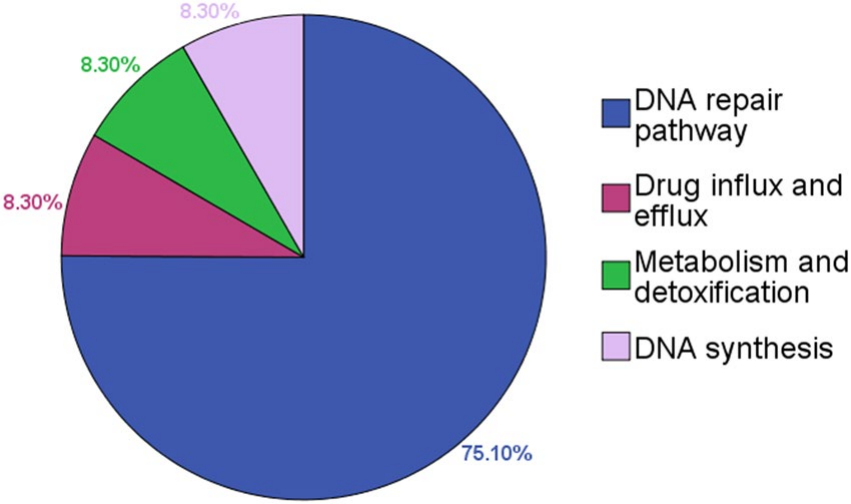
Biological pathways and physiological functions influenced by genetic variants which were statistically significantly associated with clinical outcomes of platinum-based chemotherapy in NSCLC patients.

Genetic variants significantly associated with treatment outcomes of NSCLC patients receiving PBC had impacts on several biological pathways or certain physiological functions. As shown in Fig. 2, they included DNA repair pathway (*EXCC1*, *XPD*, *XPG* and *XRCC1*), drug influx and efflux (*MDR1*), metabolism and detoxification (*GSTP1*) and DNA synthesis (*MTHFR*).

## Discussion

In this study, we described the meta-analysis findings of associations between genetic polymorphisms and treatment outcomes of NSCLC patients receiving platinum drugs. Our study identified that 14 SNPs in 10 genes were statistically associated with clinical prognosis including treatment response, OS and PFS. We further calculated FPRPs of the statistically significant results and 23 results were identified with high-quality evidence (Table 5).

The anti-cancer activity of platinum agents mainly depends on the formation of DNA adducts which inhibit DNA replication, hinder cell division and induce cell apoptosis^11^. DNA repair pathways including nucleotide excision repair (NER) and base excision repair (BER) could timely repair the damaged DNA induced by platinum agents and thus lead to treatment failure^122^. *ERCC1*, *XPA*, *XPC*, *XPD* and *XPG* are important components of NER. Being consistent with the studies by Yang *et al*.^123^ and Xu *et al*.^124^, our results confirmed the association between T allele of *ERCC1* rs11615 and shorter OS. In addition, we found that A allele of *ERCC1* rs3212986 was a risk allele that could shorten the carriers’ OS and decrease the activity of platinum, while some previously published meta-analyses did not report this effect^124–127^. However, the association should be replicated in other subsequent studies. In the present meta-analysis, we firstly assessed the influence of *ERCC1* rs2298881 variant, but no significant association was found. We studied four SNPs of XPD in this work and found that XPD rs13181, a common SNP of XP*D*, was closely related to reduced OS and PFS. For the other SNP (rs1052555) of *XPD*, we found that T allele was a risk allele and might significantly associate with unfavorable OS and PFS. This is the first meta-analysis to assess the *XPD* rs1052555 variant, and the robust association needs to be further confirmed by subsequent studies with larger sample sizes. For *XPG*, we found that rs2296147 might be related to patients’ OS, and T allele could indicate a favorable OS. The other three SNPs of *XPG* (rs1047768, rs17655 and rs873601) showed no significant association with the ORR, OS and PFS. *XRCC1* is a limiting factor in the base excision repair (BER) pathway. Our results and the previous studies confirmed the positive role of rs1799782 T allele in response to PBC^128–130^. For rs25487 of *XRCC1*, the statistically significant association between rs25487 polymorphism and ORR deserves to be further studied due to the high FRPR. *XRCC3* is also important for DNA repair, Qiu *et al*. previously reported that *XRCC3* rs861539 variation was related to good response of platinum treatment but not to survival, the same result was shown from the present meta-analysis. The *MTHFR* gene encodes an enzyme that is a central regulator for folate metabolism. It is suggested that *MTHFR* mutation was associated with increased risk of cardiovascular diseases and cancer^131^. We identified that the T allele was related to a negative response of PBC. *MDR1* gene encodes for P-glycoprotein (P-gp), which plays a major role in the process of drug efflux and influx across the cell membrane^132^. We found that *MDR1* rs1045642 variant was associated with ORR only in Asians, and published meta-analyses supported the association^133, 134^. GST is a phase II metabolic enzyme involved in the platinum detoxification, mediated by glutathione (GSH) conjugation^123^. Increasing GSH content would decrease platinum-DNA binding and result in platinum resistance. *GSTP1* gene was found to be associated with platinum treatment response, and our results indicated that T allele of *GSTP1* rs1695 increased the ORR in NSCLL patients, but the association was only observed in Asians. A previous meta-analysis also reported the same effect as ours^123^.

Great efforts have been made to identify the molecular predictive markers of platinum sensitivity. By further integrating our results according to genes biological functions, we found that the majority of polymorphisms of those genes significantly associated with treatment outcomes of platinum agents were involved in four biological pathways or physiological functions. According to the mechanism of platinum, DNA repair pathway may play a key role in the response of platinum therapy. Our results showed that the important components of DNA repair pathways (*ERCC1*, *XPD*, *XPG*, *XRCC1* and *XRCC3*) were involved in the efficacy of platinum treatment and clinical outcome of NSCLL patients. *MDR1* and *GSTP1*, which were related to drug transportation and detoxification respectively, influenced the outcome of platinum treatment. Another potential key gene was *MTHFR*, which was involved in regulating folate metabolism and DNA synthesis and was correlated with platinum sensitivity.

In the current meta-analysis, we comprehensively searched the relevant articles and explored all the eligible genes related to multiple biological functions, aiming to provide an updated and more critical summary of the available evidence of genetic polymorphisms and treatment outcomes of PBC in NSCLC patients. We first analyzed six SNPs including *ERCC1* rs2298881, *XPD* rs1052555, *XPD* rs238406, *XPG* rs17655, *XPG* rs2296147 and *XPG* rs873601. There is a high chance that an initial “statistically significant” finding based on *P* value alone turns out to be a false-positive finding, so we calculated the FPRP of each statistically significant association to ensure the credibility of our findings, and we identified 11 SNPs in 9 genes that might truly associate with the ORR and/or OS and/or PFS of NSCLC patients receiving platinum drugs.

However, there were some limits in the present meta-analysis. First, despite the intensive efforts we have made to comprehensively search the related studies, some information might have been missed. Second, between-study heterogeneity existed in the current meta-analysis. Although sensitivity analysis and subgroup analysis were applied to find the source of heterogeneity, some heterogeneity couldn’t be fully explained by statistical methods. Clinical heterogeneity might play a role in the large between-study heterogeneity, such as disease stage and age. Third, three genotypic models (heterozygote variant vs. wild type, homozygote variant vs. wild type and the dominant model) were used for this study, the other models including recessive model and allele comparison were not performed because of limited raw data. However, the models used in the study were commonly used in genetic analysis, and could in part decrease the type I error inflation^135^. Fourth, we didn’t analyze the role of gene-gene as well as gene-environment interactions in the modification of chemotherapy efficacy, and attention should be paid to these factors in further studies.

In conclusion, this collection of data might provide a useful platform for research and clinical healthy practice. Further work still needs to be done to pinpoint the use of these SNPs as prognostic biomarkers for assessing objective response and progression risk in NSCLC patients receiving platinum-based regimens.
